# Toward Development of a Vocal Fold Contact Pressure Probe: Bench-Top Validation of a Dual-Sensor Probe Using Excised Human Larynx Models

**DOI:** 10.3390/app9204360

**Published:** 2019-10-16

**Authors:** Daryush D. Mehta, James B. Kobler, Steven M. Zeitels, Matías Zañartu, Byron D. Erath, Mohsen Motie-Shirazi, Sean D. Peterson, Robert H. Petrillo, Robert E. Hillman

**Affiliations:** 1 Center for Laryngeal Surgery and Voice Rehabilitation, Massachusetts General Hospital, Boston, MA 02114, USA; 2 Department of Surgery, Massachusetts General Hospital–Harvard Medical School, Boston, MA 02114, USA; 3 MGH Institute of Health Professions, Boston, MA 02129, USA; 4 Speech and Hearing Bioscience and Technology, Division of Medical Sciences, Harvard Medical School, Boston, MA 02115, USA; 5 Department of Electronic Engineering, Universidad Técnica Federico Santa María, Valparaíso 2390123, Chile; 6 Department of Mechanical & Aeronautical Engineering, Clarkson University, Potsdam, NY 13699, USA; 7 Department of Mechanical and Mechatronics Engineering, University of Waterloo, Waterloo, ON N2L 3G1, Canada

**Keywords:** subglottal pressure, intraglottal pressure, vocal fold collision, hemilarynx, excised larynx

## Abstract

A critical element in understanding voice production mechanisms is the characterization of vocal fold collision, which is widely considered a primary etiological factor in the development of common phonotraumatic lesions such as nodules and polyps. This paper describes the development of a transoral, dual-sensor intraglottal/subglottal pressure probe for the simultaneous measurement of vocal fold collision and subglottal pressures during phonation using two miniature sensors positioned 7.6 mm apart at the distal end of a rigid cannula. Proof-of-concept testing was performed using excised whole-mount and hemilarynx human tissue aerodynamically driven into self-sustained oscillation, with systematic variation of the superior–inferior positioning of the vocal fold collision sensor. In the hemilarynx experiment, signals from the pressure sensors were synchronized with an acoustic microphone, a tracheal-surface accelerometer, and two high-speed video cameras recording at 4000 frames per second for top-down and en face imaging of the superior and medial vocal fold surfaces, respectively. As expected, the intraglottal pressure signal exhibited an impulse-like peak when vocal fold contact occurred, followed by a broader peak associated with intraglottal pressure build-up during the de-contacting phase. As subglottal pressure was increased, the peak amplitude of the collision pressure increased and typically reached a value below that of the average subglottal pressure. Results provide important baseline vocal fold collision pressure data with which computational models of voice production can be developed and in vivo measurements can be referenced.

## Featured Application

The goal of the current work is to validate a method for simultaneous measurement of vocal fold collision and subglottal pressures that can be used in vivo to better understand how collision contributes to voice disorders associated with vocal fold tissue trauma (like nodules and polyps) and to ultimately develop measures that will improve the prevention, diagnosis, and treatment of these disorders (e.g., a vocal fold collision dose).

## 1. Introduction

Many common voice disorders are believed to be primarily the result of vocal behaviors associated with voice misuse/overuse that result in vocal fold tissue trauma, or phonotrauma. Phonotrauma is typically associated with persistent vocal fold tissue inflammation, chronic cumulative tissue damage, and environmental influences that are the result of undesirable vocal behaviors that lead to the formation of benign vocal fold lesions such as nodules and polyps [1–3]. Although vocal fold impact stress (in the direction of tissue motion) and shear stress (along the tissue surface) are assumed to be critical factors in causing phonotrauma [2,4–6], there is a paucity of empirical data for these forces, including information about the actual levels that cause tissue trauma. This lack of data is largely because the in vivo measurement of vocal fold impact forces and pressures during phonation has proven challenging, and only a few published studies have been able to successfully measure vocal fold collision characteristics via intraglottally positioned devices [7–9].

Verdolini et al. [7,8] developed a piezoresistive pressure sensor with a flat frequency response up to 50 kHz and a linear sensing range of 0–14 kPa (0–140 cm H_2_O). The pressure sensing element was 1.8 mm wide × 0.4 mm thick and inserted transorally via a curved cannula. Similarly, Gunter et al. [9] developed a transorally positioned sensor housed on the tip of a curved cannula; however, the sensing element was a piezoelectric force sensor with a flat frequency response up to 25 kHz and a linear sensing range for measured forces from a 2.5 mN noise floor up to 200 mN. The force sensing element was 10 mm wide × 0.29 mm thick. For both devices, accurate positioning of the sensor in the vertical (superior–inferior) direction was critical and was accomplished using simultaneous laryngeal imaging using a rigid, transoral endoscope held in the contralateral hand of the endoscopist. As acknowledged in these studies, consistent vertical positioning is challenging due to the limitations of the two-dimensional endoscopic view of the larynx. Recordings had to be reviewed for accurate intraglottal sensor positioning, with only a small subset of recordings adequate for further analysis.

The current work sought to address the challenge of vertical placement of the vocal fold collision sensing element by incorporating an inline, dual-sensor configuration. It was hypothesized that utilizing a two-sensor probe setup would allow for the simultaneous acquisition of both vocal fold collision pressure and subglottal pressure during phonation. A distal pressure sensor would thus capture the time-varying subglottal pressure signal, while a proximal sensor would measure both the intraglottal pressure signal (aerodynamic energy during the open phase) and vocal fold collision pressure signal (impact stress during the closed phase). Directly measuring the subglottal pressure signal was expected to provide important feedback to experimenters regarding accurate vertical positioning of the intraglottal pressure sensor in real time.

This paper reports on the proof of concept of a new in vivo dual-sensor intraglottal/subglottal pressure (ISP) probe that was validated using excised whole-mount and hemilarynx phonatory models. The work follows on recently published pressure sensor characterization and validation using synthetic vocal fold models that directly informed the specifications of the in vivo probe [10]. Results from the synthetic vocal fold experiments pointed to the importance of (1) a flat pressure-sensing surface and (2) confirmation that the pressure sensor is accurately positioned to measure vocal fold contact. To validate the dual-sensor ISP probe on the bench, a hemilarynx configuration was built to enable simultaneous imaging of the superior and medial surfaces of the functional vocal fold [11,12], while also allowing for superior–inferior advancement of the pressure sensors relative to the phonating vocal fold. In this way, we may obtain a better understanding of the relationships among timing of vocal fold contact, subglottal pressure signal characteristics, and the associated features in the vocal fold collision pressure signal. Out of the scope of the current work are characterizations of prephonatory posturing (e.g., vocal process gap) and kinematic measures of the glottal cycle (e.g., open quotient). Accurate positioning of the intraglottal pressure sensor is expected to provide new insight into how collision pressure contributes to vocal fold trauma (e.g., how collision pressure varies with sound pressure level) and lead to the development of new measures that could improve prevention, diagnosis and treatment of phonotraumatic disorders. These data could also be used to help improve physical and computational models of voice production [13–17].

## 2. In Vivo Intraglottal/Subglottal Pressure (ISP) Probe

**Figure 1.**
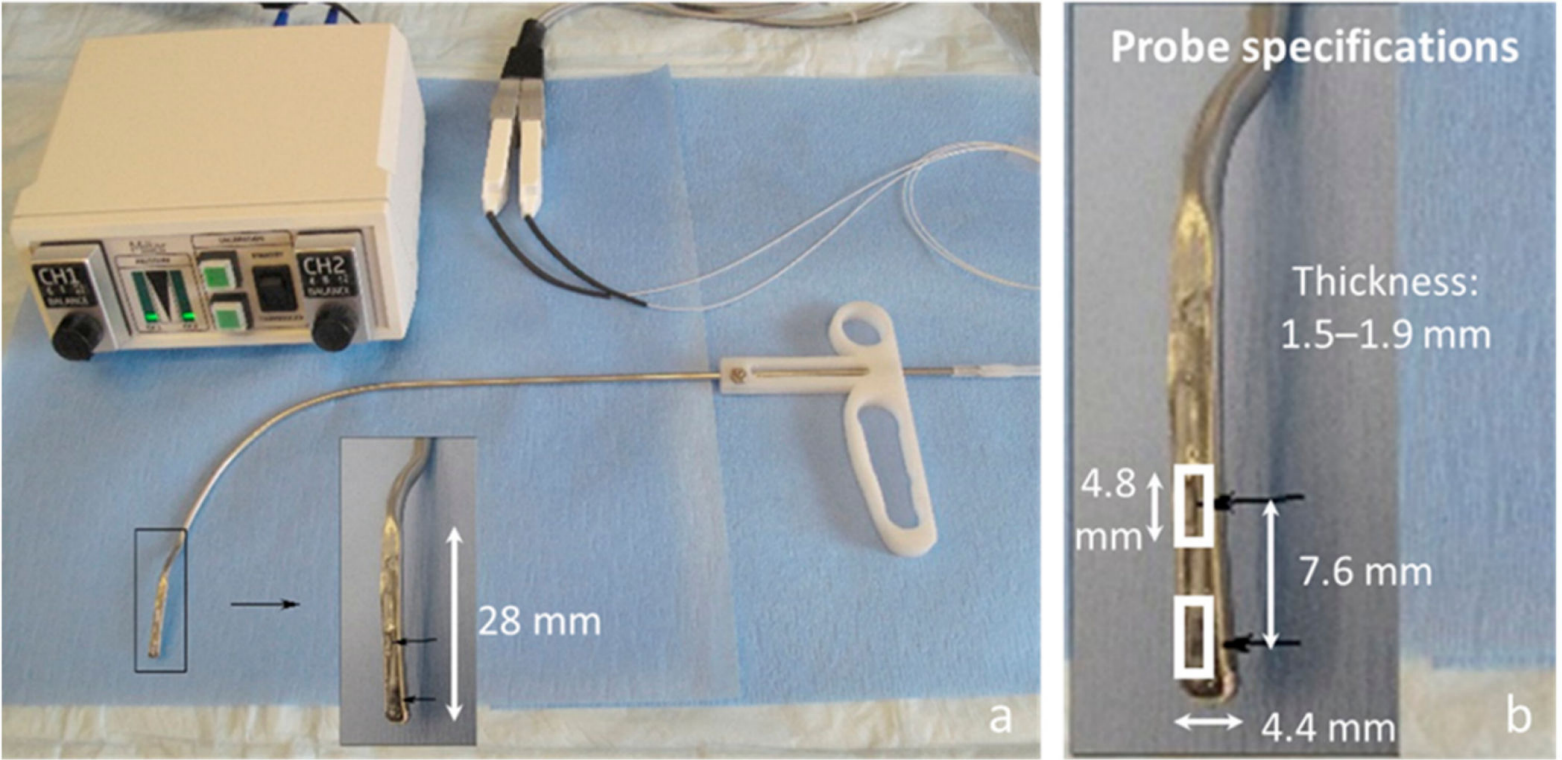
In vivo intraglottal/subglottal pressure (ISP) probe with inline, dual pressure sensors at the tip (arrows) for simultaneously measuring intraglottal and subglottal pressure during phonation. Shown are (**a**) the ISP probe with a Ford injector-like handle and two-channel signal conditioning electronics and (**b**) a zoomed-in view of the distal end of the ISP probe showing dimensions of the two inline pressure sensors embedded in epoxy.

Figure 1 displays a photograph of the dual-sensor ISP probe and its dimensions. The width of the probe tip was 4.4 mm, and the probe tip length (before curvature began) was 28 mm. The thickness of the probe at the sensor locations was maximally 1.9 mm, which is larger than in previous devices (0.4 mm [7,8] and 0.29 mm [9]) due to the dimensions of the pressure sensing elements. The larger footprint was deemed satisfactory due to the ultimate placement of the probe on a non-vibrating vocal fold of patients with a unilateral cordectomy. Simultaneous measurement of intraglottal and subglottal pressures was accomplished using two miniature pressure-sensitive sensors originally designed for heart valve and arterial pressure measurement for cardiovascular function assessment (Mikro-Cath Pressure Catheter, Millar, Inc., Houston, TX, USA). The pressure sensing elements consisted of diffused, piezoresistive semiconductors with a dynamic range of −6.7 kPa to 40 kPa (−70 cm H_2_O to 400 cm H_2_O) and a flat frequency response up to 10 kHz. The pressure sensing elements are embedded in ovoid capsules 4.8 mm long and 1.17 mm in diameter and are each connected to flexible cables 120 cm long. The two catheters are connected, via extender adapter cables, to a two-channel signal conditioning unit (PCU-2000, Millar, Inc.) that provides electrical isolation, analog knobs for zero control, and a flat frequency response of 0–1000 Hz. The ISP probe specifications are able to sense peak vocal fold impact stresses that have been reported in humans to be in the range of 0.4–3.2 kPa (4–33 cm H_2_O) [7] and subglottal pressures for loud phonation in the range of 0.6–1.5 kPa (5.9–15.4 cm H_2_O) [18]. Calibration of the pressure sensors is typically performed to account for sensor enclosures (e.g., silicone embeddings) by submerging the probe in a graduated cylinder filled with water, noting the voltage level at a given submergence depth (i.e., hydrostatic pressure) for each sensor, and computing a best-fit line to the data.

The two pressure sensors were placed inline at the exposed distal end of a custom rigid cannula whose curvature was designed to match that of a common laryngeal injector device typically used in outpatient settings for the injection of materials to medialize a paralyzed vocal fold [19] The sensors were positioned 7.6 mm apart so that the distal sensor would not touch the vocal folds (thus, measuring the aerodynamic subglottal pressure), while the proximal sensor would be positioned intraglottally to measure vocal fold collision pressures during phonation. The sensing elements of the Mikro-Cath devices employed are directional and, thus, sensitive to pressures on only one side of each capsule. We are planning to use the probes in patients who have had a hemi-laryngectomy to treat laryngeal cancer. The resulting laryngeal anatomy of these patients will allow the position of the probe to be more easily stabilized against the nonvibrating side of the glottis (mimicking an excised hemi-larynx setup) instead of trying to position the probe between two vibrating vocal folds, which has proven to be extremely challenging in previous studies (e.g., interferes with phonation) [7,9]. The ISP probe displayed in Figure 1 is a right-handed probe designed to record from patients with a left unilateral cordectomy, where the intraglottal pressure sensor comes into contact with the functioning right vocal fold during phonation. The left hand of the endoscopist is then free to operate, e.g., are endoscopic imaging system for simultaneous laryngeal visualization.

## 3. Proof-of-Concept Using an Excised Whole-Mount Human Larynx Model

### 3.1. Materials and Methods

A proof-of-concept experiment was conducted with an excised human larynx to simulate unilateral vocal fold phonation using airflow-driven oscillation at varying levels of subglottal pressure. After obtaining the tissue, the larynx was stored in a freezer at −80 °C and subsequently thawed for the current experiment. Freezing and thawing has been shown to preserve the visco-elastic properties of vocal fold tissue and allow for the assessment of biomechanics of phonation [20].

**Figure 2.**
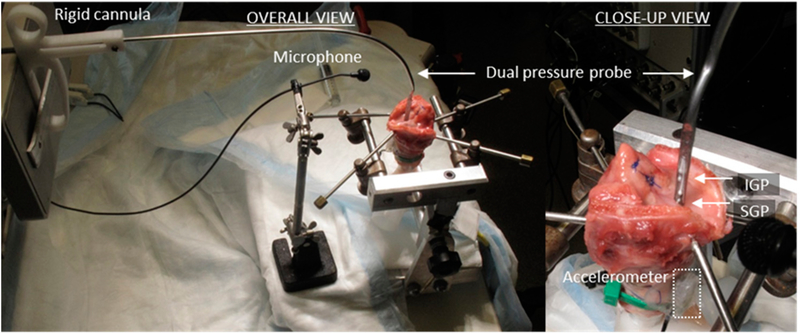
Whole-mount cadaver excised larynx preparation with ISP probe sensors measuring intraglottal pressure (IGP) and subglottal pressure (SGP) signals. An acoustic microphone was placed 15 cm from the glottis, and an accelerometer recorded the vibrations of the anterior, external tracheal wall of the specimen.

Figure 2 displays the whole-mount setup of the excised cadaver larynx that was prepared by dissecting away supraglottal structures (hyoid, etc., such that there was no supraglottal tract), suturing the ventricular folds laterally, and removing some of the superior aspect of the thyroid laminae to provide exposure of the true vocal folds. Inferiorly, the trachea was cut to a length of approximately 5 cm, and the specimen was connected to an air supply via a cylindrical tube 1.5 m in length and inner diameter of 20 mm. The larynx was mounted in a holding device that used 2 pairs of corkscrew-tipped anchoring arms to rigidly fixate the thyroid and cricoid cartilages. The arytenoid cartilages were sutured together in adduction using a single stitch passed through the bodies of the cartilages. Airflow from a medical air supply was sent through a Hudson RCI ConchaTherm III device (Teleflex, Morrisville, NC, USA) that warmed and humidified the air to 37°C before directing the air stream through the trachea to produce self-sustained vocal fold oscillations. A pneumatic pressure gauge regulated the driving pressure of the air stream, and a secondary pressure transducer acted as a reference subglottal pressure recording from approximately 6 cm below the vocal folds.

A right-handed ISP probe was positioned such that the intraglottal pressure signal measured the vocal fold collision pressure from the right vocal fold (see Figure 2). The ISP probe was placed intraglottally and pressed against the left vocal fold, effectively creating a scenario in which the left vocal fold did not vibrate when the airflow was turned on. Signals were recorded simultaneously to a data acquisition system (Digidata 1440, Molecular Devices, Sunnyvale, CA, USA) from five sensors: (1) an acoustic microphone, (2) the ISP probe’s intraglottal pressure sensor, (3) the ISP probe’s subglottal pressure sensor, (4) the secondary subglottal pressure sensor placed within the trachea in the path of the airflow (Model MPX2010GP; Motorola, Schaumburg, IL, USA), and (5) a high-bandwidth accelerometer (model BU-27135; Knowles Corp., Itasca, IL, USA). Lowpass anti-aliasing filtering was applied on all channels with a 3-dB cutoff frequency of 8 kHz (CyberAmp 380, Molecular Devices) prior to digitization at a 20 kHz sampling rate per channel. This setup enabled the measurement of vocal food collision pressures at varying levels of subglottal pressure. Future experiments may continue to investigate effects of fundamental frequency, and vocal fold adduction/abduction forces.

The use of human tissue for experimental study was approved by the Partners HealthCare System Institutional Review Board at Massachusetts General Hospital.

### 3.2 Results

**Figure 3.**
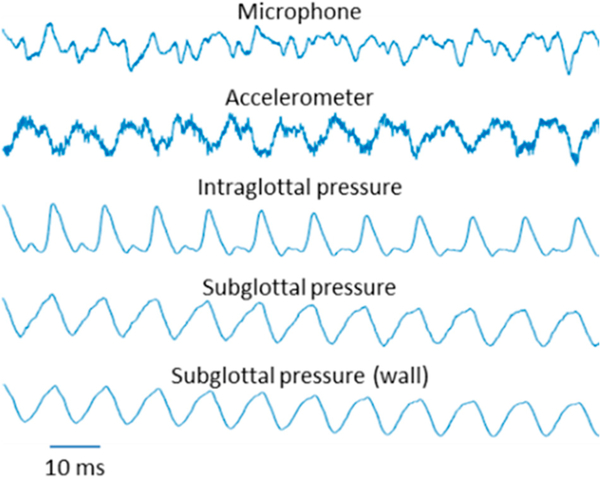
Example of synchronously recorded waveforms during self-sustained vocal fold oscillation of the whole-mount excised larynx, with ISP probe sensors measuring intraglottal pressure and subglottal pressure. Subglottal ‘wall’ pressure defers to the second pressure sensor 6 cm below the glottis.

Figure 3 displays example waveforms recorded in the whole-mount excised larynx experiment. The subglottal pressure was 3.4 cm H_2_O for a collision pressure peak of 4.5 cm H_2_O. In agreement with previous in vivo, excised, and computational studies of vocal fold impact stress, the intraglottal pressure sensor exhibited two signal components, separated in time: an intraglottal aerodynamic energy component (presumably during the open phase of the glottal cycle) and a vocal fold collision peak (presumably during the closed phase). Of interest to real-time in vivo ISP probe placement, the dual-sensor probe configuration can aid in determining correct sensor placement because the signal waveshape exhibited at the intraglottal location was differentiated from the subglottal sensor signal. Vocal fold collision is expected to be adequately captured when the intraglottal waveform exhibits an impulsive, triangular peek shape. The subglottal pressure signal measured by the ISP probe compares well with the secondary subglottal pressure sensor signal; thus, the IGP/SGP sensor spacing appears adequate so that the sensor is clear of the vocal fold tissue. The tracheal-surface accelerometer signal approximated the subglottal pressure signal waveshape, but with an added component associated with the subglottal resonances [21]. Results of the whole-mount excised larynx experiment verified that the dimensions of the ISP probe were adequate for placement in a human-sized larynx.

## 4. Importance of a Flat Pressure Sensing Surface

The ISP probe used in the whole-mount excised larynx experiment was an early prototype that embedded the Mikro-Cath pressure sensors in an epoxy that minimally covered the sensing elements of each sensor. Each Mikro-Cath pressure sensor is housed in an ovoid capsule with a recessed surface due to its primary application in a fluid environment as an arterial blood pressure sensor. When used in the present application for sensing direct mechanical contact of vocal fold tissue, inserting the embedded pressure sensor between two contacting surfaces produced difficulties in capturing the true contact pressure. Recent work using silicone vocal fold models and a ground-truth load cell has pointed to the importance of embedding each pressure sensor such that the sensing surface coming into contact with vocal fold tissue is as flat as possible for accurate pressure measurement [10]. For example, in that study, the physical size of the sensor caused the vocal fold model to deform around the sensor housing, producing a localized increase in pressure at the location of the sensor and thus over-estimating the true contact pressure. Conversely, the recessed position of the sensing element resulted in an under-estimation of the contact pressure, as the contacting material deformed down into the recessed region to contact the sensing element materials of varying stiffnesses yielded different behaviors.

The results of the silicone vocal fold model study [10] were critical in informing the improvement of the ISP probe. The ISP probe manufacturing process was revised to ensure a flat surface at the pressure-sensing end of the probe. To achieve a flat surface for the ISP probe, the sensors were embedded in medical grade room-temperature-vulcanizing (RTV) silicone and the silicone was covered with a thin 0.125 mm silicone sheet. A glass histology slide was lightly clamped against the sheet until the silicone hardened, resulting in a flat surface with the sensor elements lying immediately beneath. The added silicone layer was sufficient to fill the recessed space (air gap) and act as an appropriate surface with which the opposing vocal fold tissue in the hemilarynx model could come into contact. A flat surface above the pressure sensing element was critical to guard against significantly increased uncertainty in measurements with any recessed space or excess silicone spilling out of the space.

## 5. Excised Human Hemilarynx Experiment

In addition to incorporating a flat pressure-sensing surface, investigation of the timing of vocal fold collision relative to features in the intraglottal pressure waveform was desired to validate which waveform features related to which physiological mechanisms. The prior work with synthetic vocal fold models pointed to the potential for a pressure sensor to yield biased values for vocal fold collision pressure due to inaccurate positioning; i.e., the pressure sensor may record a peak vocal fold collision pressure that is lower than the actual peak pressure if it is not placed precisely in the strike zone during phonation [10]. In that study, both visual (high-speed video) and non-visual (electrical impedance) data served to verify that vocal fold contact occurred at the pressure sensor. To complement that work, an excised human hemilarynx experiment was designed to investigate vocal fold collision pressures during self-sustained phonation that allowed for simultaneous and synchronized high-speed videoendoscopy of the superior and medial vocal fold surfaces to aid in verifying that contact occurred at the level of the intraglottal sensor. Although limitations exist when using non-perfused laryngeal tissue and simulated phonation, the hemilarynx methodology was applied to facilitate quantitative measurements of the medial surface dynamics of the vocal folds [11]. High-speed digital imaging of the vocal folds has been applied in several studies in the literature to characterize the kinematics of the medial surface of excised human and animal tissue models [22–29], as well as during in vivo phonation [17].

### 5.1 Materials and Methods

**Figure 4.**
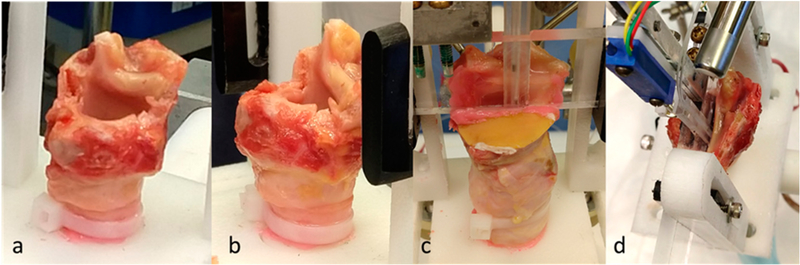
Hemilarynx preparation: (**a,b**) right laryngeal structures removed and left vocal fold and arytenoid preserved; (**c**) view of medial surface with transparent Lucite acrylic window acting as contacting surface for the left vocal fold (dental alginate affixed to seal air leaks), (**d**) view of superior surface with dovetailed slider for calibrated vertical positioning of pressure sensors.

Figure 4 illustrates the preparation of the hemilarynx that allowed for two simultaneous views (top–down and medial) during self-sustained vocal fold oscillation. Three hemilarynx models were prepared using excised tissue from adult male cadavers. In each model, the right vocal fold and associated supraglottal tissues were removed, and the specimen was mounted in a custom apparatus such that the left vocal fold vibrated against a transparent Lucite acrylic window. The window contained a narrow vertical dovetail slot machined into it, with a matching acrylic slider that could be translated up or down within the slot. A pair of Millar Mikro-Cath pressure transducers were placed 13 mm apart in the slider. The transducers were mounted in a narrow slot in the slider by embedding them in silicone with their contacting surface as flush as possible with the slider surface (similar to the design used in the in vivo probe). This setup allowed for rapid and accurate positioning of the transducers at different locations along the superior–inferior axis (e.g., one transducer within the vocal fold strike zone and the other in either a subglottal or supraglottal position). The window could also be moved and clamped horizontally so that the dovetail slider could be positioned at different locations along the anterior-posterior axis. The superior and of the dovetail slider was attached to a linear resistor, which was used in a resistor–divider circuit to track and acquire slider position during data acquisition. As in the whole-mount excised larynx experiment, a pneumatic pressure gauge regulated the air flow to the hemilarynx preparation.

**Figure 5.**
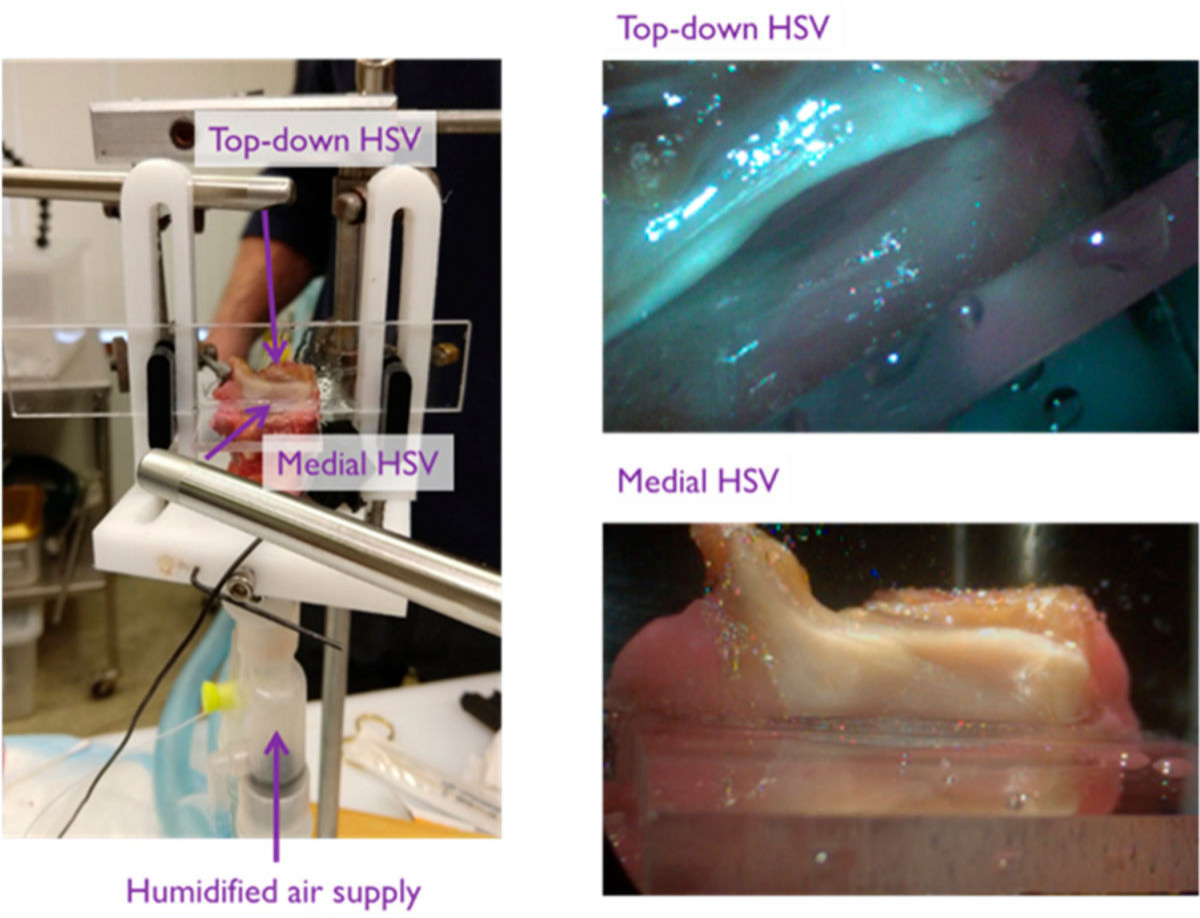
Hemilarynx preparation with two rigid endoscopies simultaneously visualizing the superior (top-down) and medial (en face) vocal fold surface using synchronized high-speed video (HSV) cameras.

Figure 5 illustrates the imaging perspectives captured by high-speed video recorded at 4000 frames per second with maximum integration time (exposure time of 248 μs). Two color high-speed cameras obtained both top-down (Phantom v7.3, Vision Research Inc., Wayne, NJ, USA.) and en face (Phantom Miro LC310, Vision Research Inc.) imaging of the superior and medial vocal fold surfaces, respectively. The spatial resolution for each camera was sea at 320 (horizontal) × 200 (vertical) pixels. Each camera was coupled to a 300 W Xenon light source by a 70° rigid endoscope with 10 mm inner diameter (JEDMED, St. Louis, MO, USA) using a 45 mm lens adapter (KayPENTAX Corp., Montvale, NJ, USA). Video S1 shows the superior surface imaging perspective overlaid on the medial perspective for an example trial.

**Figure 6.**
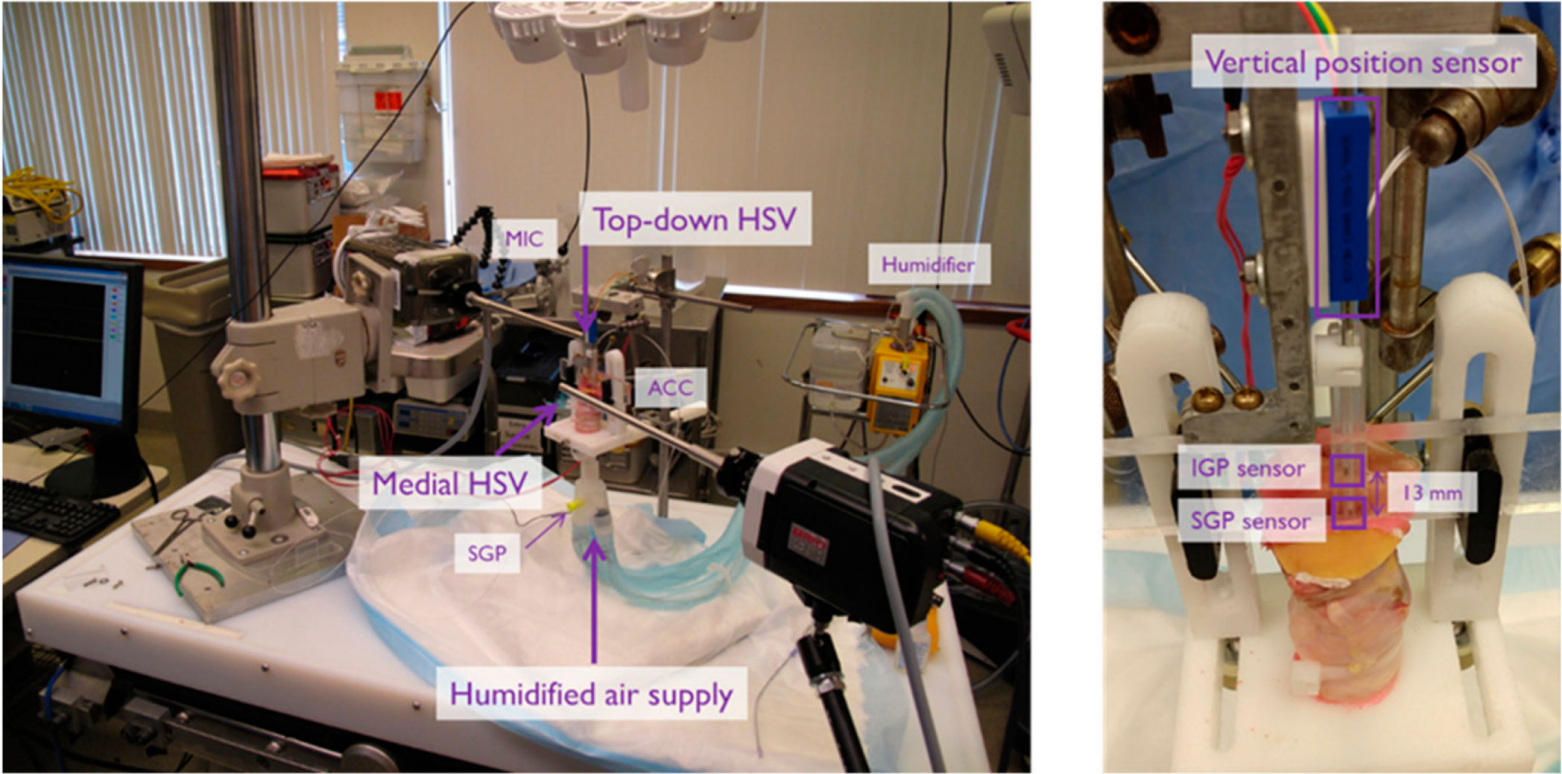
Hemilarynx experimental setup with dovetailed slider incorporating intraglottal (IGP) and subglottal (SGP) pressure sensors. IGP and SGP sensor signals were synchronized with data from the top-down high-speed video (HSV) camera, medial HSV camera, an acoustic microphone (MIC), tracheal-surface accelerometer (ACC), and a relative vertical position sensor.

Figure 6 shows a photograph of the experimental facility for the hemilarynx experiments. The video data were synchronized with five sensor signals: (1) an intraglottal pressure sensor embedded in silicon as in the in vivo probe, (2) a subglottal pressure sensor embedded in silicone as in the in vivo probe, (3) a reference subglottal pressure sensor, (4) an acoustic microphone, (5) and a high-bandwidth accelerometer mounted externally on the anterior tracheal wall. Signals were recorded simultaneously to a data acquisition system at a per-channel sampling rate of 80 kHz (Digidata 1440) with lowpass anti-aliasing filtering applied on all channels with a 3-dB cutoff frequency of 30 kHz (CyberAmp 380). The sampling rate of the two HSV cameras and sampling rate of the five channels were driven by the same master clock signal of a National Instruments data acquisition board. The hardware clock division (i.e., dividing an 80 kHz data rate into the 4 kHz video rate) and data acquisition settings were controlled by MiDAS DA software (Xcitex Corporation, Cambridge, MA, USA). For example, each video frame corresponded to 20 data samples at the frame rate of 4000 Hz. With both video and data rates derived from a common clock source, each HSV frame was synchronized with its associated data samples to within 11 μs.

The pressure sensors were embedded in medical-grade RTV silicone similar to the procedure applied to manufacture the ISP probe. The dual-sensor ISP probe was not used directly in this experiment due to tire need to use the slider, which allowed for precise and measurable positioning of the two pressure sensors in the vertical (superior–inferior) dimension using a custom vertical position sensor. As in the whole-mount excised larynx experiment, a reference third pressure transducer (bare, not embedded in any material) served as a reference measure of subglottal pressure. All pressure sensors were calibrated by submerging each sensor in a graduated cylinder filled with water and noting voltage levels at multiple submergence depths; a linear regression was fit to the data to yie1d a sensor-specific multiplicative factor that scaled voltage levels to units of cm H_2_O. The vertical position sensor was calibrated to mm, the acoustic microphone signal to pascals, and the tracheal-surface accelerometer signal to cm/s^2^.

Trials consisted of sustained vocal fold oscillation with a constant driving pressure and airflow. The superior–inferior and medial–lateral positioning of the pressure sensors were systematically varied to characterize effects on the measured vocal fold collision pressure and subglottal pressure signals. In the first set of trials, the embedded pressure sensors were moved in tandem vertically toward and past the vocal fold strike zone to mimic the sweep performed in our prior work with silicone vocal fold models (see Figure 15 in [10]). In the second set of trials, the subglottal pressure was systematically increased while one pressure sensor remained in the vertical position for measuring vocal fold collision to compare intraglottal and subglottal pressure waveforms, as well as the relative amplitude of intraglottal pressure (during the open phase of the glottal cycle) and vocal fold collision pressure (at the start of the closed phase).

A custom MATLAB graphical user interface (The MathWorks, Natick, MA, USA) was used to visualize the synchronous high-speed video and signal data in an integrated playback format [30]. The visualization allowed for frame-by-frame playback of video with linked cursors in the time-synchronized sensor signals to observe how vocal fold vibration related to acoustic and aerodynamic waveform timing at a resolution of 0.25 ms per frame. Critically, the visualization enabled the verification of the timing and location of pressure peaks associated with vocal fold contact.

**Figure 7.**
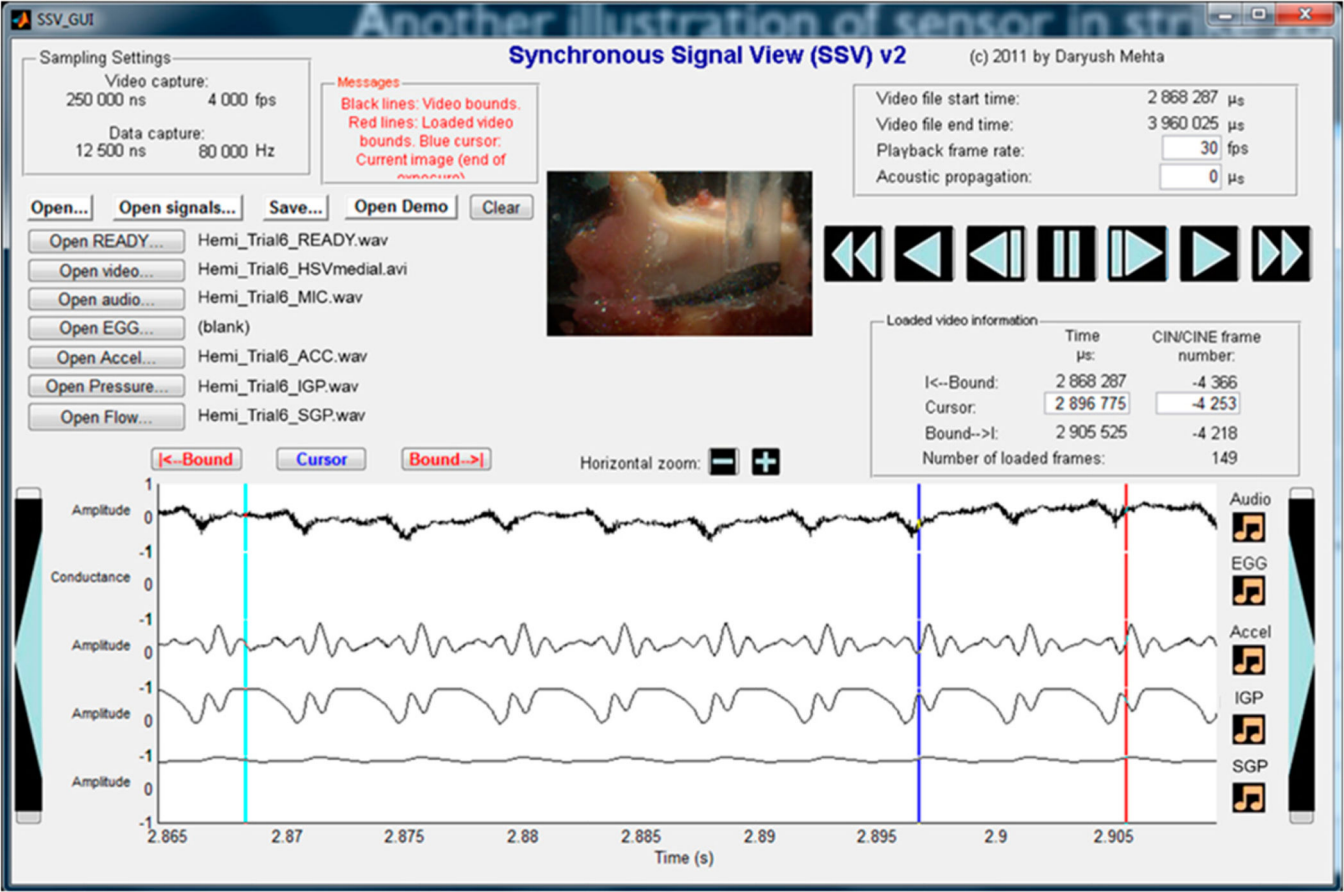
Custom graphical user interface for the playback of high-speed video data and synchronously recorded sensor signals. See Supplemental Video S2 for multimedia playback.

Figure 7 displays an example of one experimental trial using the graphical user interface. The superior surface view of the hemilarynx model was superimposed on the medial surface view to aid in validating sensor positioning and timing of vocal fold collision. Playback of the superimposed high-speed video data were then possible along with a moving cursor that indicated the corresponding time instant in the multi-sensor data channels. See Video S2 (medial view) and Video S3 (superior view) to visualize and listen to the data from this trial. Using this method of visual validation, it was verified whether the intraglottal sensor was positioned in the ‘strike zone,’ such that vocal fold contact/collision characteristics were captured. The peak collision/contact pressure (PCP) was measured from each phonatory cycle, yielding a mean PCP far a given segment. The ratio between the mean PCP (in cm H_2_O) and mean subglottal pressure (in cm H_2_O) was computed as a summary measure of vocal function for comparison with previous studies [10,12]. In vocal efficiency terms, the PCP/SGP ratio may be viewed as an indicator of power loss due to the transfer of aerodynamic power from the driving pressure and airflow to the vocal fold tissue [31,32].

### 5.2 Results

Self-sustained oscillation was achieved at mean subglottal pressures ranging from approximately 10 to 80 cm H_2_O. This range of measured subglottal pressures was significantly larger than the experimental range of mean subglottal pressures reported in prior hemilaryngeal experiments (7–44 cm H_2_O) [11,33–35] The calibration of the pressure sensors was verified. The extended upper range of subglottal pressures allowed for obtaining vocal fold collision data that potentially are associated with phonotraumatic vocal behaviors by individuals exhibiting vocal misuse and/or abuse.

#### 5.2.1 Variation in Superior–Inferior Position of the Intraglottal Pressure Sensor

**Figure 8.**
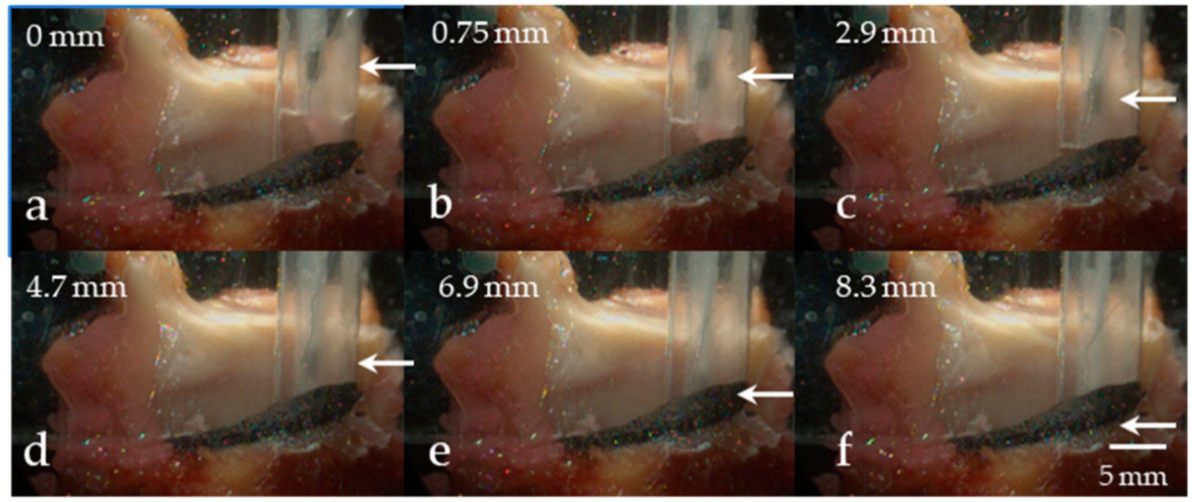
Visualization of vertical advancement of the intraglottal pressure sensor (arrow) from a reference supraglottal position (0 mm) to a subglottal position (8.3 mm) in frames from high-speed video data of the medial surface view (progression from superior to inferior position in subfigures (**a**–**f**), respectively), The pressure sensor was verified to be in the strike zone in position (**c**).

**Figure 9.**
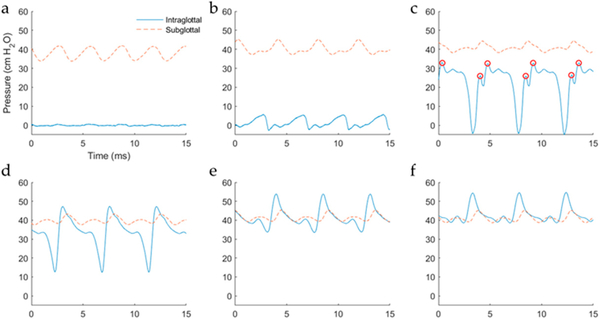
Effect of superior–inferior advancement of the intraglottal and subglottal pressure sensors. Subfigures (**a**–**f**) are from, respectively, the pressure sensor positions in subfigures (**a**–**f**) of Figure 8. The intraglottal pressure sensor was confirmed to be in the phonatory strike zone in (**c**) with vocal fold collision instants indicated (red circles).

Figure 8a–f shows images from the high-speed video data capturing the medial surface view for six experimental trials. For each successive trial, the intraglottal pressure sensor was lowered in increments of ~1–2 mm in the superior–inferior dimension. The positioning of the pressure sensor was verified from the imaging data to be positioned supraglottally in trials (a) and (b) (mean pressure of ~0 cm H_2_O), in the vocal fold strike zone in trial (c), and inferior to the strike zone in trials (d)–(f). Figure 9a–f displays exemplary phonatory cycles of the associated intraglottal and subglottal pressure waveforms for the six trials. The double peak in the intraglottal pressure waveform in Figure 9c arose due to two instants of mucosal wave collision by the lower and upper lip of the vocal fold, respectively. Following these impulsive peaks, a broader pressure peak occurred that was related to the aerodynamic intraglottal pressure build-up during the de-contacting phase of the phonatory cycle. In the subsequent trial of Figure 9d, the intraglottal pressure sensor waveform began to mirror the subglottal pressure waveshape, but with a higher peak-to-peak amplitude; imaging data in Figure 8d confirmed that the sensor was positioned inferiorly to the strike zone. Similar pressure waveform characteristics were observed in silicone hemilarynx models, where subglottal pressure measurement within 2 mm of the strike zone exhibited peaks that were not related to collision [10]. In addition, the waveform minimum did not reach 0 cm H_2_O, as is characteristic when the pressure is measured intraglottally. The intraglottal and subglottal pressure sensor waveforms in trials (e) and (f) look more and more similar as both sensor positions were located farther from the glottis in the subglottal region.

Note that the subglottal pressure waveform was expected to be similar across each of the trials; however, inputting precisely equal driving pressures was not the primary goal, and variations in tissue hydration and stiffness were possible. Of note, the fundamental frequency for each successive trial (a–f), measured from the subglottal pressure waveform, was 328, 256, 226, 217, 218, and 218 Hz, respectively. Although using excised larynx tissue may have been more challenging in terms of repeatability versus, e.g., silicone vocal fold models, advantages included more natural mucosal wave characteristics, closed phase durations, and stiffness moduli. Conclusions were not affected since both peak collision pressures and subglottal pressure were measured for each trial.

#### 5.2.2 Intraglottal Pressure Waveform Characteristics for Varying Subglottal Pressure

**Figure 10.**
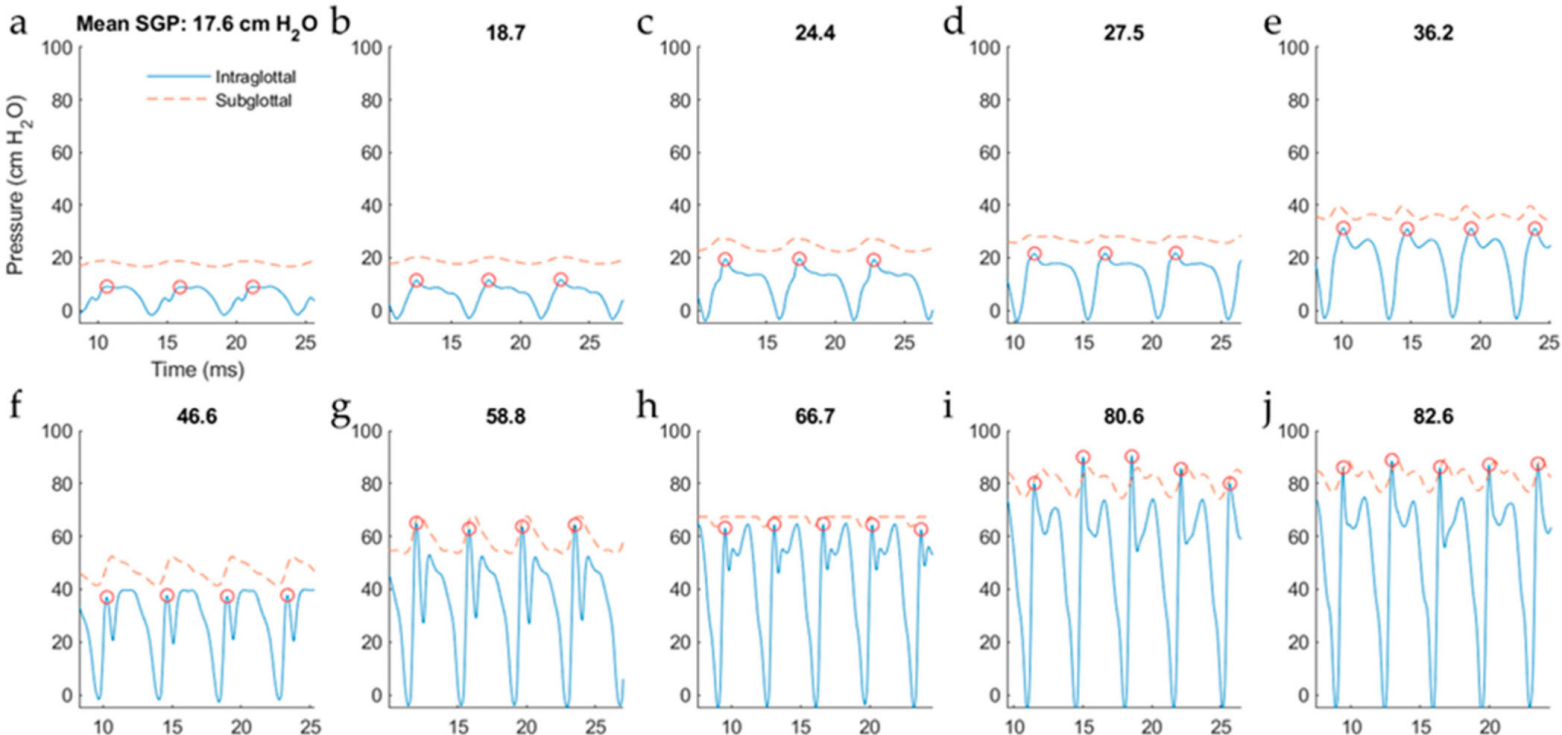
Effect of increases in mean subglottal pressure (SGP) while the intraglottal sensor position is maintained in the phonatory strike zone. Intraglottal and subglottal pressure signals are displayed for the reported mean subglottal pressures that increase from 17.6 cm H_2_O to 82.6 cm H_2_O for subfigures (**a**–**j**). Instants of peak collision pressure per cycle (red circles) are indicated in each subfigure.

**Figure 11.**
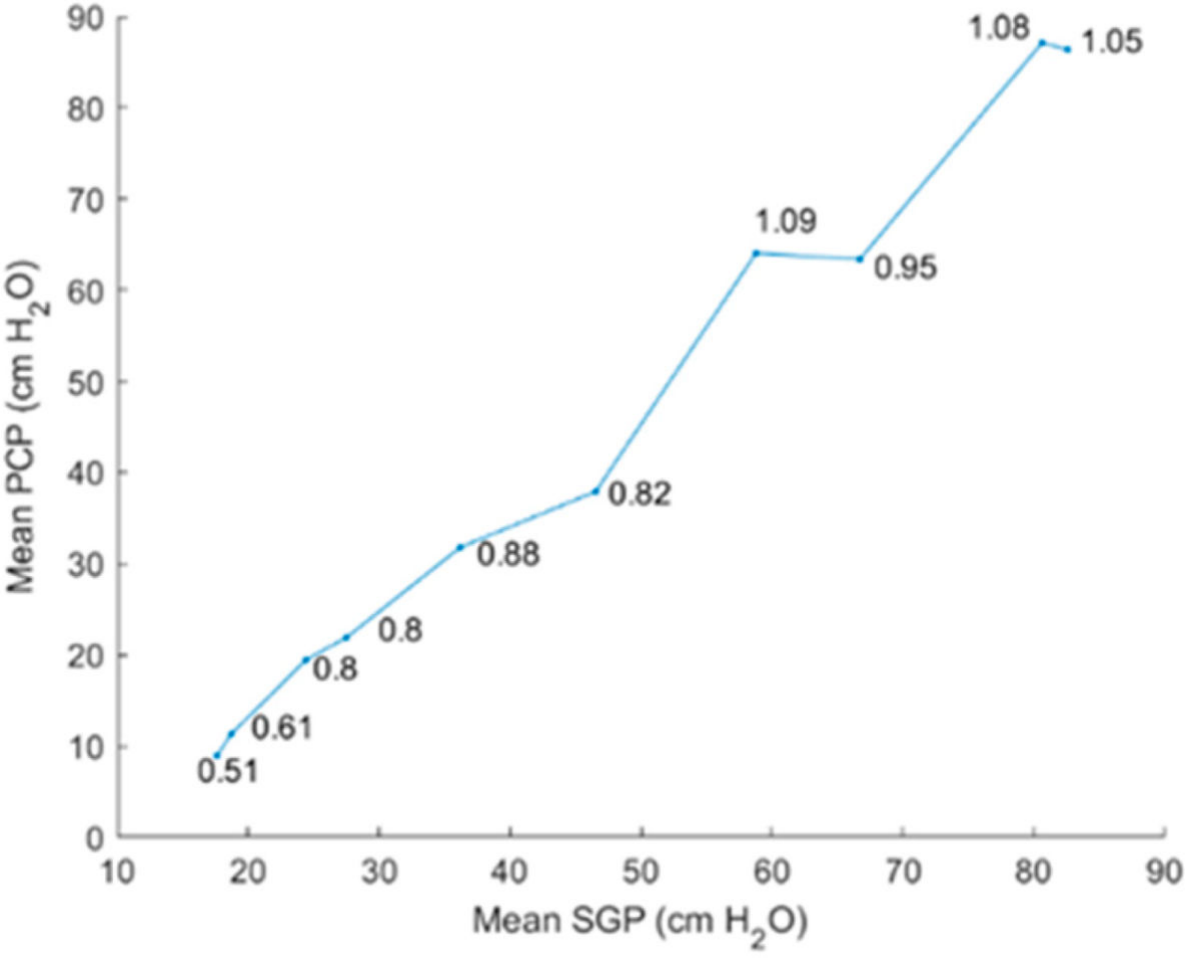
Effect of increasing subglottal pressure (SGP) on the mean peak collision pressure (PCP). Also displayed for each trial is the PCP/SGP ratio.

Figure 10 displays waveforms of the intraglottal and subglottal pressure sensors for a set of ten trials with successively increased levels of subglottal driving pressure. For these trials, the intraglottal sensor position was maintained in the phonatory strike zone such that vocal fold collision was captured by the sensor. Note that the subglottal pressure signal clipped in the eighth, with minor effect on its mean value. As expected, the classic signatures of the intraglottal pressure waveform were exhibited: (1) an impulsive pressure peak at the beginning of the closed phase, (2) a more rounded pressure peak during the de-contacting phase (still in the closed phase), and (3) a return-to-zero (or negative) pressure during the open phase of each phonatory cycle. As subglottal pressure was increased, the peak collision pressure increased and typically reached a value equal to or below that of the mean subglottal pressure; this pattern was similar to that seen in previous excised animal and computational models [33,36,37], As expected, the fundamental frequency also increased with subglottal pressure, measuring 189 Hz in the first trial to 283 Hz in the last trial. Figure 11 plots the PCP/SGP ratio for each trial. At low driving pressures, the PCP/SGP ratio is approximately 0.5, whereas PCP/SGP reaches 1 at the highest driving pressures.

## 6. Discussion

Knowledge of the expected shape of the intraglottal pressure pulse is critical to interpreting direct pressure measurements, especially as they relate to atypical phonatory mechanisms leading to voice disorders. Most bench-top studies have used canine excised larynx setups as models from which direct measures of contact pressures have been obtained and correlated with other measures such as the electroglottography-based contact quotient [38] and HSV-derived vocal fold tissue acceleration [33,39]. Researchers continue to explore indirect methods to estimate vocal fold contact pressures to facilitate this process in vivo [16,17,36]; but efforts in that direction require further validation with direct contact pressure measurements.

### 6.1 Comparison of Results to Prior Work

Prior attempts at measuring in vivo vocal fold collision pressure using a single sensor have yielded mixed results, including uncharacteristic intraglottal pressure waveshapes with no impulsive peak signature [9] or impulsive pressure signatures without information regarding sensor positioning or concomitant subglottal driving pressures [7]. In the current work, a dual-sensor ISP probe was developed to mitigate issues related to the uncertainty in sensor positioning. Incorporating multiple pressure taps in physical models of phonation is common [37], and adding a second sensor to the in vivo ISP probe was a natural step to provide important information to the endoscopist regarding subglottal pressure characteristics. Emphasis was placed on characterizing the intraglottal and subglottal pressure waveshapes using a human hemilarynx model such that these waveform properties can then be translated to measurement in the human in vivo setting.

In agreement with prior work that employed animal hemilarynx models [12,37], silicone vocal fold models [10,36], and numerical modeling [40], the intraglottal pressure signal in the current human excised larynx study exhibited both contact/collision and aerodynamic components separated in time. In particular, sweeping the vertical (superior–inferior) position of the pressure sensor from a supraglottal to a subglottal location was performed to mimic the experimental protocol of [10]. In that study, the pressure sensor position was swept in increments of 0.5 mm from 2 mm above to a mm below a silicone hemilarynx model. Results of that work indicated that measurement of peak collision pressure may exhibit underestimation of up to 20% relative to the true collision pressure when the sensor was as close as 0.5 mm above or below the strike zone. This underestimation was hypothesized to be due to the sensor simultaneously capturing both impact and aerodynamic pressures. In addition, the pressure waveform at the offset location was similar to that in the strike zone such that one would not know of the offset sensor position without independent verification. Similar results were observed in the current study when the pressure sensor was swept from above the glottis to a subglottal location (see Figures 8 and 9). However, knowledge of the simultaneous recorded subglottal pressure waveform enabled immediate feedback as to any differences from the intraglottal pressure waveform. Another telling signature that the pressure sensor was in the strike zone was a strong return-to-zero-pressure property of the negative-going peak.

In the hemilarynx experiment, the PCP/SGP ratio was approximately one at higher subglottal driving pressures, with PCP/SGP decreasing to half that value at lower driving pressures (Figure 11). The PCP/SGP ratio has been reported in a previous excised canine hemilarynx experiment to range from 1.1 to 3.8 [12]. Recent work employing a silicone hemilarynx model exhibited a PCP/SGP ratio of 1.15, with medial compression properties of the laryngeal configuration having a significant impact on the ratio [10]. Further work is needed to understand the variables (mode of vibration, rheometric tissue properties, etc.) associated with the conversion of aerodynamic power to vocal fold collision.

### 6.2 ISP Probe Considerations in Practice

Since measuring vocal fold collision pressures during bilateral vocal fold phonation has proven to be challenging in vivo [7,9]. Positioning a pressure probe against one vocal fold in a normal larynx can result in very disordered phonation due to tissue anterior and posterior to the probe vibrating in a chaotic fashion, which also disrupts vibration (and pressure contact measurements) on the contralateral side due to the irregular glottal closure characteristics. These issues are mitigated in a select group of patients with laryngeal cancer who have undergone a conservative endoscopic treatment that allows for subsequent voice restoration [41–43]. Following -this surgical procedure (a partial laryngectomy), one intact vocal fold comes in contact with a surgically reconstructed, non-vibrating scar band on the contralateral side that is medialized to promote glottal closure. The scar band replaces the vocal fold that has been surgically removed to treat the cancer. Many of these patients have perceptually normal conversational voices compared with the pre-surgical condition of a glottal insufficiency producing an excessively inefficient and breathy voice In these patients, the ISP probe would rest against a patient’s non-vibrating vocal fold to yield stable and reliable collision pressure measurements of the medial surface of the contralateral vocal fold, under the assumption that proper anesthetic of the laryngeal tissues would not alter basic phonatory mechanics.

**Figure 12.**
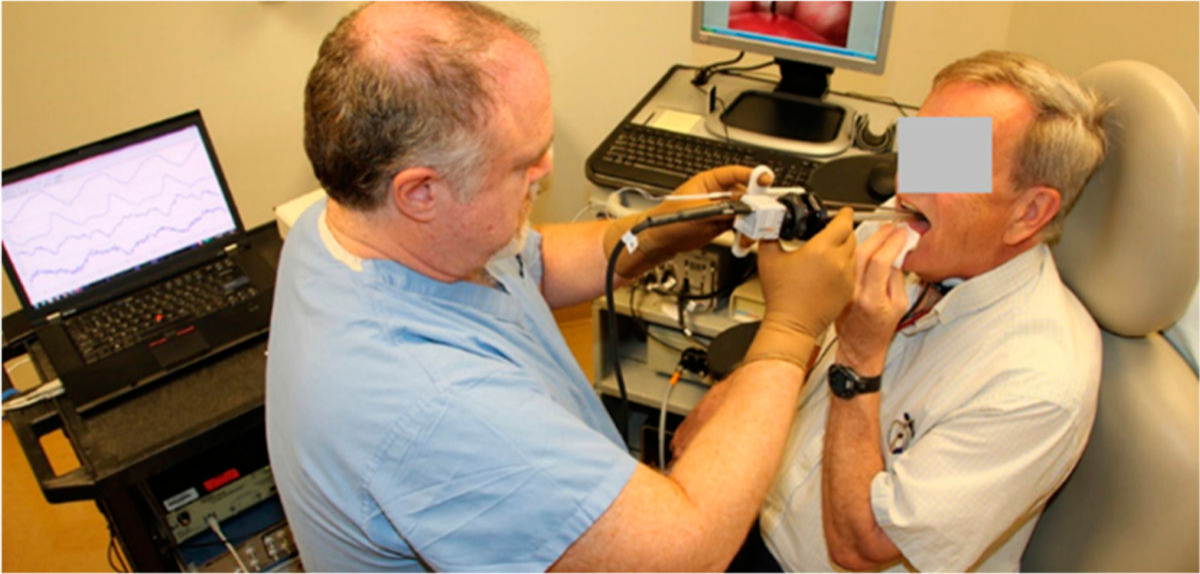
Illustration of a clinician holding a left-handed ISP probe that is designed to measure the left vocal fold collision pressure. The clinician’s right hand operates a standard camera sensor for standard video or video stroboscopic imaging.

Figure 12 illustrates the ISP probe configuration in which the clinician holds a left-handed ISP probe with pressure sensing elements facing the left vocal fold of the patients with a right unilateral cordectomy. In the photograph, the clinician’s right hand holds a transoral rigid endoscope coupled to a laryngeal endoscopic imaging system to visualize the vibrating vocal fold in real time. This kind of transoral laryngeal procedure, which is performed using topical anesthesia of the upper airway, has been a routine part of the laryngeal surgeon’s practice for several decades. As with any office-based procedure that places an instrument in proximity to the vocal cords (injections, biopsies, etc.), the surgeon must be skilled with office-based laryngeal techniques. The risk of mild irritation/abrasion of tissue during the pressure measurements is minimized by using a cannula that does not have any sharp edges.

In practice, the following guidelines are recommended for maintaining appropriate positioning of the ISP probe to accurately capture vocal fold collision characteristics:

1. Endoscopic visualization of the larynx during phonation via videostroboscopy or high-speed videoendoscopy for general placement of the ISP probe such that the proximal sensor is positioned intraglottally and the distal sensor subglottally.
2. In patients with a unilateral cordectomy, placement of the ISP probe on the non-vibrating flap of tissue to maintain a flat surface on which the contralateral, functioning vocal fold can come into contact.
3. Real-time monitoring of both pressure sensor waveforms to verify that the two waveforms are distinct from each other. In particular, the proximal pressure sensor would be in the strike zone during phonation when the intraglottal pressure waveform exhibits:
  - An impulsive peak in the direction of increasing pressure at the start of the closed phase;
  - A rounded peak following the impulsive peak during the open phase;
  - A minimum value reaching a zero or negative value during the open phase.

It may be necessary to ask the subject to produce a sustained vowel while the ISP probe is swept in the superior–inferior dimension due to challenges in precise positioning. During data analysis, the features above can be tracked over time to determine when adequate vocal fold contact occurred to accurately capture peak collision pressures.

### 6.3 Implications for Vocal Dose Measures

A critical element in the development of ambulatory vocal dose measures is the characterization of vocal fold collision [44], which is widely considered to be a primary etiological factor of phonotrauma. In their original formulation, vocal dose measures were designed to indirectly qualify the accumulated effects of rapid acceleration and deceleration of vocal fold tissue (distance dose) and the breaking of molecular bonds at the cellular level due to thermal agitation (energy dissipation dose), drawing from occupational standards limiting the vibratory amplitude, duration, and frequency of hand tools [1]. Further development of vocal dose measures incorporated measures of impact stress/collision pressure on vocal fold tissue during vocal fold vibration [44]. The inclusion of collision/impact stress is very important in any quantification or modeling of phonotrauma since vocal fold nodules are widely considered to form due to repetitive stress on the mid-membranous portion of the vocal folds [2,4,6]. The tracheal-surface accelerometer position in the excised larynx experiments mimics the subglottal placement of ambulatory accelerometers positioned on the anterior neck surface of human subjects [45]. Future investigation of the relationship between vocal fold impact stress/collision pressure and the tracheal-surface accelerometer signal is hypothesized to enhance ambulatory voice monitoring technology. Measures of vocal fold collision doses that can be implemented in ambulatory voice monitoring and biofeedback systems could be enhanced to represent the accumulation of vocal fold collision pressures over long periods of time while individuals go about their typical daily vocal activities.

## 7. Conclusions

A dual-channel probe was developed to simultaneously measure intraglottal pressure and subglottal pressure signals for patients with a unilateral cordectomy to gain empirical insight into vocal fold collision and aerodynamic relationships during phonation. The dual-sensor configuration enables the simultaneous capture of intraglottal pressure and subglottal pressure to aid in the separation of the components of the intraglottal pressure sensor signal into an aerodynamic energy component (during the open phase) and vocal fold impact stress component (during the closed phase). The excised hemilarynx experimental setup provided important baseline vocal fold collision pressure data with which computational models of voice production can be developed and in vivo measurements can be referenced. A long-term goal of this work is to continue developing ambulatory vocal dose measures that incorporate vocal fold collision information and can be estimated from noninvasive neck-surface vibration signals.

## Supplementary Material

Video S1

Video S2

Video S3
